# Commentary: Sustaining and scaling a clinic-based approach to address health-related social needs

**DOI:** 10.3389/frhs.2025.1571843

**Published:** 2025-05-12

**Authors:** Bo Kim

**Affiliations:** ^1^ Center for Health Optimization and Implementation Research, VA Boston Healthcare System, Boston, MA, United States; ^2^ Department of Psychiatry, Harvard Medical School, Boston, MA, United States

**Keywords:** commentary, quality improvement, sustainability, contextual factors, implementation science

A Commentary on Sustaining and scaling a clinic-based approach to address health-related social needs By Arbour M, Fico P, Floyd B, Morton S, Hampton P, Murphy Sims J, Atwood S and Sege R (2023). Front. Health Serv. 3:1040992. doi: 10.3389/frhs.2023.1040992

## 1 Introduction

Scaling and sustaining evidence-based interventions (EBIs) in healthcare is a persistent challenge, particularly for models addressing health-related social needs (HRSN). Arbour et al. contribute valuable insights through their study of the Developmental Understanding and Legal Collaboration for Everyone (DULCE) model, which integrates social needs screening into pediatric well-child visits (WCVs). Their study evaluates whether a lower-intensity continuous quality improvement (CQI) strategy can sustain and expand DULCE while preserving key outcomes.

By analyzing the transition from intensive to lower-intensity implementation support, this study highlights strategies for maintaining EBIs with fewer resources. These findings align with broader efforts to integrate social care into healthcare while balancing scalability and cost-effectiveness (1). This commentary expands on Arbour et al.'s findings by discussing CQI's role in sustainability, the influence of local context, and equity considerations in implementation support.

## 2 Continuous quality improvement as a strategy for sustainability

A key strength of Arbour et al.'s study is its examination of CQI as a strategy for sustaining EBIs. Their findings suggest that a lower-intensity CQI model – featuring quarterly coaching calls rather than frequent, individualized support – was largely effective in maintaining intervention fidelity and HRSN screening rates. However, WCV adherence varied, indicating that different intervention components may require varying levels of support.

This raises a crucial implementation science question: how much support is necessary for sustainability? Research highlights the need to balance fidelity and adaptation (2), and this study provides insight into how reduced support affects different outcomes. While CQI offers a structured approach to sustainability, further research should determine the optimal frequency and intensity of CQI activities.

Additionally, workforce engagement is central to sustainability. Family Specialists in DULCE are critical to connecting families with social services, and frontline provider engagement has been linked to intervention success (3). Understanding how CQI impacts staff retention and workload could help refine implementation strategies. Future studies should examine whether variations in CQI design influence provider satisfaction and burnout, both of which affect sustainability (4).

## 3 Contextual factors and variability in sustainability

A key insight from the study is the variation in intervention sustainability across sites. While some clinics maintained or improved outcomes under the lighter-touch CQI model, others saw declines, particularly in WCV adherence. This suggests that clinic-specific factors – such as leadership support, staffing capacity, and healthcare infrastructure – play a crucial role in sustainability.

These findings align with research emphasizing that organizational readiness, leadership engagement, and contextual fit influence implementation success (5). Arbour et al.'s study suggests that while some settings may sustain interventions with reduced support, others may require ongoing or more intensive assistance. Future research should examine how site characteristics influence implementation strategies and how to tailor support accordingly.

Additionally, organizational culture plays a role in intervention sustainability (6). Clinics with established quality improvement cultures may be better equipped to sustain EBIs, even with reduced external support. Identifying how different organizational factors interact with CQI efforts could help refine sustainability strategies across diverse healthcare settings.

## 4 Equity considerations in implementation and scale-up

The study raises important equity considerations, as DULCE was implemented in clinics serving primarily Medicaid-enrolled, historically marginalized families. By embedding social needs screening within pediatric care, the model has the potential to reduce health disparities. However, variability in WCV adherence under the lighter-touch CQI model suggests that reductions in support may have uneven effects across different clinics.

Research has emphasized that equity-focused implementation strategies must address structural barriers to sustainability (7). If certain clinics require more intensive support, strategies should be flexible to prevent reductions in support from exacerbating disparities. Policy-level interventions – such as Medicaid reimbursement for social needs screening – could help sustain these models in under-resourced settings (8).

Additionally, integrating an equity lens into CQI processes is essential. Rather than assuming all clinics can sustain interventions with the same level of reduced support, strategies should assess site-specific challenges related to provider capacity and community resources. A more tailored CQI approach – such as targeted coaching for clinics facing greater barriers – could help mitigate disparities in implementation outcomes.

Future studies should also investigate whether patient population characteristics influence the effectiveness of lighter-touch CQI models. Research suggests that interventions addressing social needs require adaptations based on contextual factors such as transportation access, language barriers, and available social services (9). A stratified CQI approach, informed by clinic and community characteristics, may optimize sustainability while advancing health equity.

## 5 Future directions in implementation science

Building on Arbour et al.'s findings, several key areas for future research emerge. First, more research is needed to determine how much CQI support is necessary to sustain EBIs without diminishing effectiveness. Second, given the focus on social determinants of health, future studies should examine how alternative payment models (e.g., Medicaid waivers, value-based care) can support long-term sustainability. Third, understanding organizational and contextual factors influencing sustainability can help develop adaptive implementation strategies that provide varying levels of support based on clinic needs. Fourth, future work should explore whether reductions in implementation support disproportionately impact under-resourced clinics and identify strategies to ensure equitable intervention sustainability. Fifth, examining how different CQI models affect staff retention, burnout, and job satisfaction could refine strategies for long-term implementation.

## 6 Discussion

Arbour et al.'s study provides valuable insights into the feasibility of using a lighter-touch CQI approach to sustain a clinic-based social needs intervention. The findings contribute to ongoing discussions in implementation science about balancing fidelity and flexibility, understanding contextual influences on sustainability, and addressing equity considerations in intervention scale-up.

As implementation science continues to refine strategies for sustaining EBIs, this study underscores the importance of tailoring implementation support to local needs. Ensuring that effective interventions remain impactful over time – particularly in historically marginalized communities – will require continued innovation in implementation strategies, alongside policy and practice-level efforts that prioritize sustainability and equity.
